# Everybody's Page

**Published:** 1889-01-12

**Authors:** 


### Everybody's Page.

The Fever Hospital at Manchester was founded in May, 1796, when an asylum, containing from fifteen to twenty-five beds, was thought large enough for the town's necessities.

In 1887 the death-rate of Florence was 24·47 per 1,000 inhabitants, or 4,993 persons in a population of 174,947. Of these deaths 768 were due to zymotic diseases and 688 to consumption.

The third finger of the hand—the ring finger—was selected for the honour of wearing the wedding ring because formerly it was believed that a nerve passed directly from it to the heart.

A hair-dresser near the Palais Royal, in Paris, advertises "Callileucapillaire water, to dye the hair white, for the use of young physicians and magistrates." He should invent a wash for inducing baldness when he is about it.

A member of the French Senate has suggested that poisoning by prussic acid would be a good method of inflicting capital punishment, while a still more tender-hearted gentleman advises a pleasant enthauasia by means of chloroform.

The latest thing in inoculations is buffalo-pox. In India they are cultivating the bovine virus for vaccination through young buffaloes. But, shades of Mayne Reid and Fenimore Cooper, is this a romantic use to make of the "lion of the ruminants" that roamed over the prairies of our youth?

It sounds rather strange to hear of a case of belladonna poisoning caused by the wearing of a bella donna plaster when the skin was a little broken, but such a case has occurred. The treatment which cured the symptoms of poisoning was repeated doses of sulphate of morphine and bromide of potassium.

The decrease in the consumption of alcohol which can now be chronicled, is somewhat counterbalanced by the increased indulgence in narcotics which is commonest, unfortunately, among the educated classes. This vice is not so prevalent here as in America, where it is proposed to erect an asylum solely for those addicted to the use of opium, chloral, and cocaine.

In March, 1800, the Rev. J. T. A. Reed began the practice of vaccination in two parishes, Leckhampstead and Akeley, near Buckingham. His doing so surprised the people, for, though they all admitted that nobody had ever died of cow-pox, and that nobody had ever had small-pox after it, they thought it odd that anybody should wilfully contract the milder disease. He succeeded, however, in getting 500 patients to submit, and their immunity in the midst of an epidemic of small-pox induced 1,000 more to come forward and seek vaccination before going to the great fair held on May Day at Towcester, where they feared the infection of small-pox.

Should a physician ever whip his patient? This was the question brought up for decision at a Berlin police-court some time ago. A doctor was asked to prescribe for a boy four years of age, who was suffering from some slight ailment, but the child screamed so violently that it was impossible to examine him. After trying for a long time to soothe the child the physician resorted to the old-fashioned method of giving him something to cry for, and boxed his ears. The child's mother not only resented this, but showed her resentment in a practical manner by summoning the doctor for assault, but the Court decided that the medical man had acted only for the patient's good, and so acquitted him.

A federation has been formed in Paris for the cheapening of medical attendance. The federation propose to pay two francs a-quarter for adults, and one franc for children under sixteen. This is cheap enough in all conscience. A large number of medical men have received invitations to become members of the medical staff of this union; but it is not stated how many have accepted the attractive invitation.

In February, 1797, a man, a smith by trade, was sent to the Middlesex House of Correction, for being concerned in a fraudulent transaction into which he had been drawn by some shrewder villains. During his year's imprisonment he mended all the locks of the prison, and did other smith's work, the value of which was estimated at nearly £170. When his time of detention was up he was given £30 out of the produce of his labour, and he then said that he had been so kindly treated by the governor of the gaol, that if he could maintain his wife and children there he would gladly pass his life there. Accordingly, he was appointed county smith, and his wife inspectress of female prisoners, and they spent their lives happily within the precincts of the gaol.

Dr. Howard Pinkney is of opinion that a turpentine bath is good for rheumatism, gout, insomnia, laryngitis, and bronchitis, and from his own experience recommends the following method of preparing it: Make a saturated solution of six ounces of yellow soap, and add to it three or fours ounces of oil of turpentine. Shake the contents well before they are put into the bath, which should be filled with warm water. The result is a pleasant bath, from which exhales the genial odour of pine, and which causes a glow all over the body. After fifteen minutes' immersion, the patient should be put to bed, where he will probably soon fall into a refreshing sleep.

It takes about five men to form a firm of quack medicine manufacturers. First, there is the literary man, who writes the letters with the narratives of marvellous cures; next comes the artist, who shows the patient thin before and plump after taking the medicine (or *vice versâ* if the cure of obesity be the object desired); then there is the poet, who celebrates the merits of the drug in verses that won't scan; the liar who has been cured (of nothing) a score of times by using the special decoction to be puffed; and the forger who writes testimonials from all parts of the country to the same effect. These are the important men; anybody can do the mixing of quassia and water, or whatever the "safe cure" or "infallible specific" consist of.

While milk has long had the eye of suspicion fixed upon it as the possible bearer of infection, butter has hitherto escaped suspicion. Yet it has dangers of its own. Butter is often made in farmhouse kitchens in which the whole family live, and to which, in case of illness, convalescents are carried, as being the warmest room in the house. If the milk or cream that is to be churned is in the kitchen, it will, under such circumstances, readily absorb disease-germs, and other and more direct means of conveying infection are not far to seek. An American doctor says: "I have seen the mother, who was the housekeeper and only nurse, leave a large wooden bowl full of butter, in which her hands and arms had been immersed almost to the elbows, and follow me through two adjoining rooms in which her children were sick with scarlet fever, give me the history of their illness the night previous, receive directions and instructions, and return to her butter-packing without stopping to wash her hands. And this butter was sought by local dealers, shipped to City markets, where well-watched and carefully-isolated innocents developed those mysterious *de novo* cases of scarlet fever.

